# Evaluating competency and influential factors among infection control nurses in South Korean healthcare settings

**DOI:** 10.1017/ash.2025.127

**Published:** 2025-09-03

**Authors:** Choi Jongrim

**Affiliations:** College of NursingKeimyung University, Daegu, Republic Of Korea

**Keywords:** Competency, Infection Control Nurse, Influencing factors

## Abstract

**Objectives:** The competency of infection control nurses (ICNs) plays a pivotal role in enhancing the quality of healthcare facilities. This descriptive study aimed to assess the competency level of ICNs in South Korea and identify influencing factors. **Method:** An online self- administered questionnaire survey was conducted through an announcement on the Korean Infection Control Nurses Association website. An online self-administered questionnaire was distributed via the Korean Infection Control Nurses Association website, garnering responses from 199 participants out of 450 approached. Statistical analyses, including descriptive statistics and multiple regression analysis, were conducted using SPSS/WIN 27.0 software. **Result:** Analysis reveals that participants had an average age of 34.8 (±8.4) years and an average of 5.0 (±4.7) years of experience in infection control. The competency score for infection control was 3.6 out of 5. Competency levels varied across domains, with the highest scores observed in employee safety and infection control domains, while the lowest scores were in infectious disease identification and communication domains. Significant variables affecting competency, as identified through univariate analysis, included awareness of infection control competency, age, education level, ICN experience, position, and possession of an infection control specialist license. Ultimately, factors influencing ICN competencies were determined to be awareness of infection control competencies, attainment of a master’s degree or higher, over 5 years of ICN experience, and age over 50, collectively explaining 45.6% of the variance. **Conclusion:** Enhancing the competency of ICNs is crucial for effective infection control in medical settings. Strategies to improve awareness of infection control competencies and provision of continuous education support and career development programs for ICNs are essential to achieve this goal.

